# Epidemiology of paediatric Middle East respiratory syndrome coronavirus and implications for the control of coronavirus virus disease 2019

**DOI:** 10.1111/jpc.15014

**Published:** 2020-07-30

**Authors:** Chandini R MacIntyre, Xin Chen, Dillon C Adam, Abrar A Chughtai

**Affiliations:** ^1^ Biosecurity Program, The Kirby Institute, Faculty of Medicine University of New South Wales Sydney New South Wales Australia; ^2^ School of Public Health and Community Medicine, Faculty of Medicine University of New South Wales Sydney New South Wales Australia

**Keywords:** asymptomatic infections, communicable diseases, emerging, COVID‐19, Middle East respiratory syndrome coronavirus, Saudi Arabia

## Abstract

**Aim:**

To compare the clinical features of Middle East respiratory syndrome coronavirus (MERS‐CoV) infection between paediatric and adult cases.

**Methods:**

Using multiple public data sources, we created an enhanced open‐source surveillance dataset of all MERS‐CoV cases between 20 September 2012 and 31 December 2018 in Saudi Arabia including available risk factor data.

**Results:**

Of the 1791 cases of MERS‐CoV identified, 30 cases (1.7%) were aged under 18 years and 1725 cases (96.3%) were aged 18 years and over. Three paediatric cases were fatal, aged 0, 2 and 15 years. The odds of asymptomatic MERS‐CoV infection among cases under 18 years (*n* = 10/23; 44%) was significantly higher (odds ratio (OR) = 4.98; 95% confidence interval (CI): 2.15–11.51; *P* = 0.001) compared to adults (*n* = 199/1487; 13%). The odds of hospitalisation were significantly lower (OR = 0.17; 95% CI: 0.08–0.39; *P* < 0.001) among cases under 18 years (*n* = 12/24; 50%) compared to adults (*n* = 1231/1443; 85%). Children were more likely to have a known source of exposure compared to adults (OR = 2.68; 95% CI: 1.29–5.56; *P* = 0.008).

**Conclusions:**

Clinically severe illness is less common in children, although death can occur, and the proportion of paediatric cases (1.7%) is similar to that reported for COVID‐19. Age‐specific differences in the clinical presentation of MERS‐CoV cases could have implications for transmission for other betacoronaviruses including severe acute respiratory syndrome coronavirus 2 (SARS‐CoV‐2). Children may be at risk within the household with an infected adult. More studies are required on the role of children in transmission of betacoronaviruses.

## What is already known on this topic

1. Asymptomatic infection with coronaviruses including MERS‐CoV and SARS‐CoV‐2/COVID‐19 has been frequently reported, especially in children and young adults, but age‐specific differences are poorly described.
2. Paediatric cases of MERS‐CoV have been described in a small case series.

## What this paper adds

1. Children infected with MERS‐CoV were more likely to present with asymptomatic infection compared to adults and less likely to be hospitalised, but may still have fatal outcomes.
2. Children are more likely to have a known source of exposure than adults, indicating they may acquire infection from an adult household contact.
3. These results could have implications for the control of MERS‐CoV and other betacoronaviruses such as SARS‐CoV2/COVID‐19.

The Middle East respiratory syndrome coronavirus (MERS‐CoV) is a positive‐sense, single‐stranded RNA virus^1^ which was first isolated from an infected human case in Bisha, Saudi Arabia in 2012.^2^ MERS‐CoV viral RNAs have been isolated from dromedary camels in several Middle Eastern and African countries, and are now thought to be the natural reservoir host for MERS‐CoV,^3^ although a spill‐over event from bats has been suggested.^4^ The exact source of MERS‐CoV emergence into the human population, however, remains unknown. The epidemiology of MERS‐CoV shows a predominance among males, older persons, and those with chronic diseases particularly affected.^5^ During the severe acute respiratory syndrome (SARS) outbreak in 2002, an increased risk for males and older adults was also described^6^ with less severe illness observed among children under the age of 12 years.^7^ There are few studies comparing MERS‐CoV in adults and children. A case series of 31 paediatric cases of MERS‐CoV found only 3 were asymptomatic, most had mild respiratory illness, 2 had pneumonia on chest radiograph and 1 required ventilation, but there were no deaths.^8^ With the emergence of SARS‐CoV‐2/Coronavirus Virus Disease 2019 (COVID‐19) in late December 2019, there has been increasing interest in the epidemiology of paediatric cases and the potential for asymptomatic infection. For example, a child within a family of confirmed cases of SARS‐CoV‐2/COVID‐19 was found to have the ground glass appearance of pneumonitis on a chest computed tomography scan despite being asymptomatic.^7^ Furthermore, in a larger case series of 99 hospitalised SARS‐CoV‐2/COVID‐19 patients the youngest was 21 years old, suggesting younger adults and children are not often hospitalised.^9^ Potential reasons for this could be that children present with mild illness or are more likely to be asymptomatic. Additionally, in a cohort study of 72 314 SARS‐CoV‐2/COVID‐19 cases in China, it was found that 8.1% of cases were under the age of 20 years, and 1.2% of the total cohort was asymptomatic.^10^ In a retrospective study of 2135 children under 18 years with SARS‐CoV‐2/COVID‐19 in China, only 4.4% were asymptomatic cases, and 30% had moderate to severe disease, 6% were critical and there was one death.^11^ Another study found that 15% were asymptomatic, 41% had fever and the median age was 6.7 years.^12^ The clinical features of symptomatic children include acute upper respiratory tract infection such as fever, cough, sore throat and sneezing, pneumonia, accompanied with diarrhoea, and may progress to respiratory failure and multiply organ dysfunction.^11^ In the USA, 1.7% (2572/149 082) of cases were children, with a median age of 11 years.^13^ Fever was less common, 5.7% were hospitalised, 2% required intensive care and three deaths occurred.

There appears many common features between MERS and SARS coronavirus^14^; however, comprehensive age‐specific epidemiology, with a comparison of risk factors between adults and children is not available. We aimed to analyse the comparative epidemiology of paediatric and adult cases of MERS‐CoV in Saudi Arabia from 2012 to 2018.

## Methods

### MERS‐CoV enhanced epidemiological dataset

We created an enhanced surveillance database using open source data, comprising all human cases of MERS‐CoV infection reported from 20 September 2012 to 31 December 2018 in Saudi Arabia. Data including age, gender and location were sourced from case lists from the Saudi Ministry of Health national events reports,^15^ World Health Organization disease outbreak news,^16^ and Novel Coronavirus MERS Announced Cases data set available from FluTrackers.^17^ We crosschecked data between linked reports from the World Health Organization^16^ and Saudi Ministry of Health^15^ for each human case and enhanced the dataset with clinical and disease factor data, including date of notification, date of symptoms onset, date of hospitalisation, laboratory confirmation date, disease complications and contact history. Symptoms and outcomes, including death were also included where available. Paediatric cases were defined as cases in people aged <18 years. We excluded 113 cases where data was unavailable for all risk factors.

### Statistical analysis

Descriptive epidemiologic analysis was conducted. Statistical differences between gender, asymptomatic rate, hospitalisation rate, death rate, the rate of complications and the rate of exposure to confirmed cases by age groups <18 years and ≥18 years were analysed using the Pearson's χ^2^‐test. Odds ratios and 95% confidence intervals were calculated using logistic regression. All reported *P* values were based on the logistic regression. A *P* value<0.05 was considered statistically significant. We used the SPSS Statistics v22 (IBM Corp., Armonk, NY, USA) for statistical analysis.

## Results

**Fig 1:**
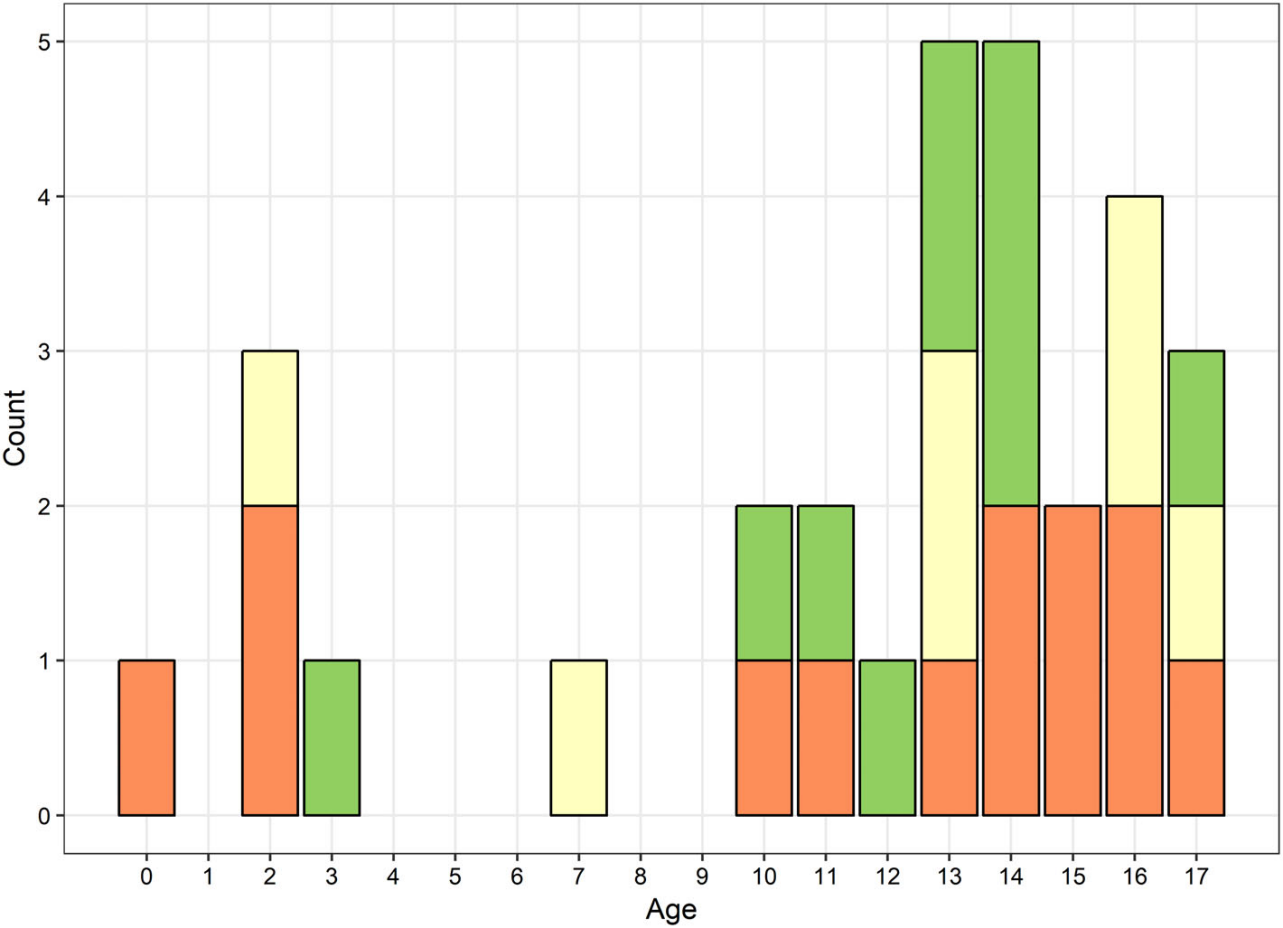
Age distribution of 30 paediatric cases of Middle East respiratory syndrome coronavirus in Saudi Arabia by clinical presentation 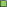 Asymptomatic; 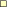 Unknown; 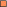 Symptomatic.

Of 1791 cases in the database, 30 cases (1.7%) were aged under 18 years and 1725 cases (96.3%) were aged 18 years and over. Details on age were missing for the remaining 36 cases. The mean age of cases under 18 years was 11 years, with a range 0–17 years. Figure 1 shows the age distribution of cases aged under 18 years by clinical presentation ‐ asymptomatic cases were spread across all ages. Among the 30 paediatric cases, 16 were male, 12 female and 2 of unknown gender. Three paediatric cases were fatal, aged 0, 2 and 15 years. Hospitalisation was recorded in 50% of paediatric cases (*n* = 12/24; six missing) and 43% were asymptomatic (*n* = 10/23; seven unknown). No further details were available on asymptomatic cases. Complications were reported in 33% of paediatric cases (*n* = 10). No paediatric cases had exposure to animals, and 43% had exposure to confirmed human cases. Most paediatric cases occurred in Riyadh (*n* = 12; capital and largest city of 4.2 M+), followed by Jeddah (*n* = 5; second largest city of 2.8 M+), Dawmat Aljandal (*n* = 4; remote) and three or less each in Madinah, Hafr Al‐Batin and Al Jawf Region.

The odds of asymptomatic MERS‐CoV infection among children was significantly higher (*P* < 0.001) compared to adults (Table 1). Furthermore, the odds of hospitalisation due to MERS‐CoV (*P* < 0.001) and complications (*P* = 0.001) were significantly lower among children compared to adults (Table 1). Children were more likely than adults to have exposure to a known case (*P* = 0.008) (Table 1). There were no significant differences in gender or mortality rate (*P* = 0.206 and 0.158, respectively) by age group (Table 1).

**Table 1:** Comparison of paediatric and adult cases of Middle East respiratory syndrome coronavirus in Saudi Arabia from 2012 to 2018

Variable	Paediatric, *n* (%)	Adult, *n* (%)	OR (95% CI)	*P* value
Male	16/28 (57)	1168/1706 (68.5)	0.61 (0.29–1.31)	0.206
Asymptomatic	10/23 (43)	199/1487 (13.4)	4.98 (2.15–11.51)	<0.001
Hospital admission	12/24 (50)	1231/1443 (85.2)	0.17 (0.08–0.39)	<0.001
Death	3/30 (10)	359/1723 (20.8)	0.42 (0.13–1.40)	0.158
Complication	10/30 (33)	1061/1618 (66)	0.26 (0.12–0.57)	0.001
Exposure to confirmed cases	13/30 (43)	383/1723 (22)	2.68 (1.29–5.56)	0.008

CI, confidence interval.

## Discussion

Of all notified cases of MERS‐CoV, paediatric cases are rare (<2% of all observed cases) but can be fatal. We found 1.7% of cases were children, which is the same rate of paediatric cases described for COVID‐19.^12^ Cases were geographically dispersed however 40% (12/30) occurred in the capital and largest city Riyadh. In our study, we found that asymptomatic cases are significantly more common in children compared to adults, and MERS‐CoV is significantly less likely to result in the hospitalisation of children. Children were more likely to have a known source of exposure than adults, which may indicate that children are more likely to acquire infection in the household from an infected adult. Testing of all close contacts cases of MERS‐CoV, regardless of symptoms and including children, may be important.

There have been some reports of asymptomatic transmission of MERS‐CoV^18^ particularly among hospital‐associated outbreaks^18^ and of SARS‐CoV‐2/COVID‐19 including prior to symptom onset.^19^ Together with our results, this suggests that children could represent a source of undetected betacoronavirus infection. The testing of only symptomatic contacts may fail to limit spread, if there is substantial asymptomatic or mildly symptomatic infection in children. The rate of MERS‐CoV infection among children may be even higher than observed in notified data, as asymptomatic close contacts are less likely to be tested. Community testing of children during outbreaks of MERS‐CoV or testing of all children with close contact histories to infected cases may shed more light on the epidemiology of MERS‐CoV in children. Viral shedding data on infected children would also be informative. Prolonged viral shedding for up to 27 days in children, especially in the faeces, has been described with SARS‐CoV‐2.^20^ There are no similar studies published for MERS‐CoV.

The limitations of this study could be the bias in results due to inability to obtain detailed clinical data, the small number of paediatric cases and missing data. However, we enhanced the surveillance data of all the reported human cases in Saudi Arabia over a 6‐year period, with available risk factor data, which provided a comprehensive comparison of MERS‐CoV infection among paediatric cases and adults.

Understanding the comparative epidemiology of paediatric and adult cases has important implications for the outbreak management and diagnosis of MERS‐CoV and other betacoronaviruses. Further studies are required to inform our understanding of MERS‐CoV epidemiology, including serological and viral shedding studies and the role of paediatric cases in community transmission. Diagnostic procedures could include routine sequencing of isolates which could be used to identify specific molecular risk factors for mild or asymptomatic infection in the future. SARS‐CoV, MERS‐CoV and SARS‐CoV‐2/COVID‐19 all preferentially cause severe disease in older adults, but the role of mildly symptomatic or asymptomatic children in transmission is unknown and understudied.

## Conclusion

Severe illness of MERS‐CoV appears less common in children, and the proportion of paediatric cases (1.7%) is similar to that reported for COVID‐19. Howeverm fatal cases do occur in children. Age‐specific differences between the clinical presentation of paediatric and adult cases of MERS‐CoV could have implications for transmission. Children may be at higher risk of infection within households from an infected adult, but could be asymptomatic. More studies are required on the role of children in transmission of betacoronaviruses.
